# Carbolized Potash as an Obtunder

**Published:** 1884-12

**Authors:** 


CARBOLIZED POTASH AS AN OBTUNDER.—Dr. J. A. Robinson has recently suggested the use of equal parts of caustic potase and carbolic acid as an obtunder for sensitive dentine. In cases of exposed pulp the application of this compound, it is claimed, produces but slight pain, an eschar being soon formed, which will admit of a capping of oxyphosphate of zinc. What the subsequent effect upon the vitality of the pulp will be, time only can determine. It is prepared by rubbing up in a mortar equal parts by weight of carbolic acid, in crystals and caustic potash, until a crystative paste is formed. It has also been successfully employed in the treatment of pyorrhœa alveolaris, applied on floss silk or cotton fibres around the necks of the teeth, and pressed by means of a small flat burnisher into the pockets in the gum between and about the margins of the teeth. Dr. Robinson claims that an experiment of three years in an extensive practice, has demonstrated the value of this new compound. Dr. Geo. Watt is of the opinion that as phenol sodique is a combination of carbolic acid with caustic soda, this combination of carbolized potash may prove even mere useful as a disinfectant and antiseptic.

